# Retrospective analysis of the Draize test for serious eye damage/eye irritation: importance of understanding the in vivo endpoints under UN GHS/EU CLP for the development and evaluation of in vitro test methods

**DOI:** 10.1007/s00204-013-1156-8

**Published:** 2013-12-28

**Authors:** Els Adriaens, João Barroso, Chantra Eskes, Sebastian Hoffmann, Pauline McNamee, Nathalie Alépée, Sandrine Bessou-Touya, Ann De Smedt, Bart De Wever, Uwe Pfannenbecker, Magalie Tailhardat, Valérie Zuang

**Affiliations:** 1 Adriaens Consulting BVBA, Bellem, Belgium; 2 Institute for Health and Consumer Protection, European Commission Joint Research Centre, Ispra, VA Italy; 3 The Procter & Gamble Company, Egham, Surrey, UK; 4 L’Oréal Research & Innovation, Aulnay Sous Bois, France; 5 Laboratoire Pierre Fabre, Castres, France; 6 Janssen Research & Development, Beerse, Belgium; 7 Henkel AG & Co. KGaA, Düsseldorf, Germany; 8 Beiersdorf AG, Hamburg, Germany; 9 LVMH Recherche, St. Jean De Braye Cedex, France; 10 Present Address: Services & Consultation on Alternative Methods Sagl (SeCAM), Agno, Switzerland; 11 Present Address: seh consulting + services, Paderborn, Germany

**Keywords:** UN GHS/EU CLP, Drivers of classification, Eye irritation/serious eye damage, Draize within-test variability, Validation of in vitro methods

## Abstract

For more than two decades, scientists have been trying to replace the regulatory in vivo Draize eye test by in vitro methods, but so far only partial replacement has been achieved. In order to better understand the reasons for this, historical in vivo rabbit data were analysed in detail and resampled with the purpose of (1) revealing which of the in vivo endpoints are most important in driving United Nations Globally Harmonized System/European Union Regulation on Classification, Labelling and Packaging (UN GHS/EU CLP) classification for serious eye damage/eye irritation and (2) evaluating the method’s within-test variability for proposing acceptable and justifiable target values of sensitivity and specificity for alternative methods and their combinations in testing strategies. Among the Cat 1 chemicals evaluated, 36–65 % (depending on the database) were classified based only on persistence of effects, with the remaining being classified mostly based on severe corneal effects. Iritis was found to rarely drive the classification (<4 % of both Cat 1 and Cat 2 chemicals). The two most important endpoints driving Cat 2 classification are conjunctiva redness (75–81 %) and corneal opacity (54–75 %). The resampling analyses demonstrated an overall probability of at least 11 % that chemicals classified as Cat 1 by the Draize eye test could be equally identified as Cat 2 and of about 12 % for Cat 2 chemicals to be equally identified as No Cat. On the other hand, the over-classification error for No Cat and Cat 2 was negligible (<1 %), which strongly suggests a high over-predictive power of the Draize eye test. Moreover, our analyses of the classification drivers suggest a critical revision of the UN GHS/EU CLP decision criteria for the classification of chemicals based on Draize eye test data, in particular Cat 1 based only on persistence of conjunctiva effects or corneal opacity scores of 4. In order to successfully replace the regulatory in vivo Draize eye test, it will be important to recognise these uncertainties and to have in vitro tools to address the most important in vivo endpoints identified in this paper.

## Introduction

The serious eye damage/eye irritation test developed by Draize et al. (1944) has been for over half a century the reference method for ocular hazard identification. However, several in vitro methods for assessing the serious eye damage/eye irritation potential of chemicals have been developed as alternatives to the Draize eye test. Currently, only three methods are adopted by the Organisation for Economic Co-operation and Development (OECD) as partial replacements to classify chemicals as inducing serious eye damage (UN GHS/EU CLP Category 1). These are two organotypic assays, the Bovine Corneal Opacity and Permeability (BCOP) test method (OECD TG 437) and the Isolated Chicken Eye (ICE) test method (OECD TG 438) (OECD 2013a, b), and a cell-based assay, the Fluorescein Leakage (FL) test method (OECD TG 460) (OECD 2012b). In addition, two of these methods (BCOP and ICE) were recently adopted by the OECD for the identification of chemicals not requiring a classification for serious eye damage/eye irritation (UN GHS/EU CLP No Category) (OECD 2013a, b). Two other test methods, namely the Cytosensor Microphysiometer (CM) (Hartung et al. 2010) and the Short-Time Exposure (STE) (Takahashi et al. 2008, 2009; Sakaguchi et al. 2011), are currently under the process of regulatory acceptance by the OECD for the identification of both UN GHS/EU CLP Category 1 and No Category chemicals, with limited applicability domains (ESAC 2009; ICCVAM 2013). However, no single in vitro method or combination of methods has yet been successfully validated for full replacement of the in vivo Draize eye test (Scott et al. 2010).

Accurate analyses of the in vivo Draize eye test data are important for several reasons. The outcome of validation studies depends not only on the performance of the in vitro method but also on the quality and variability of the in vivo data that usually serve as a reference point for comparison, in the absence of standardised human data. Chemicals that do not have reliable in vivo data of sufficient quality should not be selected as reference chemicals. It is therefore imperative that the quality of the in vivo data is carefully assessed, so that a robust selection of candidate chemicals for validation studies can be made. The impact of the variability of the in vivo Draize eye test, including inter alia the “in vivo within-test variability”, on the final classification of a chemical is another important issue for the selection of reference chemicals. With increasing variability, the uncertainty about the result increases. Therefore, it is not desirable to select chemicals with highly variable in vivo results to benchmark data in the validation of in vitro methods. Moreover, the determination of the most relevant in vivo endpoint(s), in particular the effects on cornea, iris or conjunctiva, is extremely important for the development of adequate in vitro methods and will allow better understanding of the relationship between the in vitro and the in vivo data. Such an analysis can also provide valuable information on the usefulness and limitations of the in vitro assays, for example by clarifying which ocular endpoints the alternative methods are able to predict. Ultimately, a thorough analysis of animal reference data allows taking into account the imperfectness of accepted reference test methods in the assessment of new alternative test methods developed to replace them.

The present study reports a detailed statistical analysis and resampling of historical in vivo Draize eye test data, performed with the purpose of (1) determining the most important tissue effect(s) (corneal opacity, iritis, conjunctiva chemosis, conjunctiva redness) for the classification of chemicals in vivo according to the United Nations Globally Harmonized System of Classification and Labelling of Chemicals (UN GHS) (UN 2013) and the European Union Regulation on Classification, Labelling and Packaging of chemicals (EU CLP) implementing UN GHS in the EU (EC 2008), (2) determining the importance of irreversibility of ocular effects for classification of chemicals in vivo (e.g. proportion of chemicals classified on the basis of persistence of effects), (3) evaluating the test method’s within-test variability based on the current data interpretation procedures, and (4) proposing acceptable and justifiable target values of sensitivity and specificity for alternative methods and their combinations in testing strategies. The historical in vivo Draize eye test data used in this work originated from several sources including many industry sectors, and no new in vivo data were generated to perform the analyses. These data were obtained from (1) three Reference Chemicals Databases (RCD) containing data mostly on “existing chemicals” (on the EU industrial market before 1981) and (2) from the European New Chemicals Database (NCD) of the ex-European Chemicals Bureau (ex-ECB) containing data on “new chemicals” notified under Directive 67/548/EEC (introduced to the EU industrial market after September 1981). The RCD contains commercially available chemicals that have been commonly used in validation studies of in vitro methods and for which full in vivo Draize eye test data are available. The NCD, on the other hand, contains proprietary chemicals that have not been commonly used in validation studies, includes more complex chemistries and contains only summarised in vivo Draize eye test data. Since data in RCD and NCD may inherently differ, potentially with confounding factors, separate analyses were performed on these two sets of data.

## Materials and methods

### Databases

The in vivo rabbit eye irritation data were collected from data registered in the NCD of the ex-ECB and three Reference Chemicals Databases (RCD), i.e. (1) the Eye Irritation Reference Chemicals Data Bank (ECETOC) (Bagley et al. 1992a, 1999a; ECETOC 1998), (2) the database from ZEBET (Spielmann et al. 1996), and (3) the database from Laboratoire National de la Santé (LNS) (Gautheron et al. 1992). Before starting exploratory data analysis, a quality check of the Draize eye test data was performed. Studies were excluded from the final analysis if (1) the study criteria allowing an unambiguous classification were not met (SCNM), i.e. studies of less than 21 days where full reversibility (scores equal to zero) of all endpoints in all animals did not occur and no severe effects were observed, (2) less than three animals were used and no severe and/or persistent effects were observed, (3) data were incomplete, i.e. missing data for at least one endpoint or no individual data accessible (NCD *n* > 3 animals), or (4) classification in the original database occurred without a tissue score above the threshold value or persistence of effects being noted and where the reason of classification was not indicated (chemicals classified in the absence of a classification triggering effect) (Table 1).

**Table 1:** Draize eye studies from various data sources

Data source	Number of studies			UN GHS/EU CLP (proportion of valid studies)		
	Total	Excluded^a^	Valid	No Cat	Cat 2	Cat 1
RCD 1—ECETOC	149	12	137	56.2	18.2	25.5
RCD 2—ZEBET	143	55	88	61.4	14.8	23.9
RCD 3—LNS	52	3	49	69.4	18.4	12.2
RCD—Total	344	70	274	60.2	17.2	22.6
NCD—Total	2,319	459	1,860	82.6	10.4	6.9

*RCD* Reference Chemicals Databases, *NCD* European New Chemicals Database

^a^(i) if study criteria allowing an unambiguous classification were not met (SCNM), (ii) if less than three animals were used and no severe and/or persistent effects were observed, (iii) in case of incomplete data or (iv) in case chemicals were classified in the absence of a classification triggering effect (i.e. not based on a second (*n* = 3 or 4) or third (*n* = 5 or 6) highest animal mean tissue score above the classification threshold or on persistence of an effect) and the reason of classification was not indicated

### Draize eye test method

The historical data from the RCD and NCD were generated with albino rabbits according to the OECD Test Guideline 405 (OECD 2012a). Briefly, a single dose (100 μl volume for liquids and solids) of the test material was placed into the conjunctiva cul-de-sac of the eye of the animal. Solid chemicals in the LNS database and at least the ones tested after 2002 in the NCD were rinsed from the eye 1 h after application with saline or distilled water when the solids were still present (according to the 2002 revision of OECD TG 405, even though LNS predates this revision), while solid chemicals in the ECETOC and ZEBET databases were not (these chemicals were tested according to OECD TG 405 prior to its 2002 revision). Subsequently, the eyes were observed at selected time intervals for up to 21 days for signs of ocular lesions. For each RCD and NCD chemical, the following endpoints driving classification were determined and evaluated: corneal opacity (CO), iritis (IR), conjunctiva chemosis (CC), conjunctiva redness (CR), and days to clear. Scores for lesions were given from 1 to 2 for IR, from 1 to 3 for CR, and from 1 to 4 for CO and CC. A score of 0 was given to a tissue when no lesions were observed in that tissue.

### UN GHS and EU CLP classification of the RCD and NCD chemicals

The UN GHS and EU CLP classification of chemicals tested in albino rabbits according to the Draize eye test method (OECD 2012a) is primarily based on the severity of effects and timing of reversibility of effects. According to the UN GHS and EU CLP classification systems, Category 1 is defined as causing irreversible effects on the eye/serious damage to the eye and Category 2 as causing reversible effects on the eye/eye irritation. The classification rules defined by UN GHS (UN 2013) and EU CLP (EC 2008) and the ECHA Guidance on the application of the CLP criteria for classification of studies with more than three animals (ECHA, 2012; chapter 3.3.2.3.2.2 In vivo data) were used to classify all the chemicals (from individual studies) included in our analyses. An overview of the classification rules is presented in Table 2. First, mean CO, IR, CC, and CR scores over the reading times at 24, 48, and 72 h were calculated for each animal. Next, the second highest (for studies with 3 or 4 animals) or the third highest (for studies with 5 or 6 animals) of the mean CO, IR, CR, and CC scores (CO^Maj^, IR^Maj^, CR^Maj^ and CC^Maj^) were determined for each study to examine if the majority of the animals (i.e. 2 out of 3, 3 out of 4, 3 out of 5 or 4 out of 6 animals) had a mean tissue score less than, equal to or greater than the defined threshold value(s) for classification. An UN GHS/EU CLP Category 1 (Cat 1) was assigned based on CO^Maj^ ≥ 3 and/or IR^Maj^ > 1.5 and/or CO = 4 (observed at any time point in any rabbit during the observation period, before day 21) and/or a persistent effect (score >0 on day 21 on any tissue in any animal). An UN GHS/EU CLP Category 2 (Cat 2) classification was assigned based on 1 ≤ CO^Maj^ < 3 and/or 1 ≤ IR^Maj^ ≤ 1.5 and/or CR^Maj^ ≥ 2 and/or CC^Maj^ ≥ 2. If none of the criteria for Cat 1 and Cat 2 defined above were met, the chemical does not require classification for serious eye damage/eye irritation, and therefore, No Category (No Cat) was assigned.

**Table 2:** Classification rules defined by UN GHS (UN 2013) and EU CLP (EC 2008)

Endpoint	Range scores^a^	Category 2^b^	Category 1^c^
		Reversible effects on the eye/eye irritation	Irreversible effects on the eye/serious damage to eyes
CO	0–4	1 ≤ CO^Maj^ < 3; and/or	CO^Maj^ ≥ 3; and/or
IR	0–2	1 ≤ IR^Maj^ ≤ 1.5; and/or	IR^Maj^ > 1.5
CR	0–3	CR^Maj^ ≥ 2; and/or	
CC	0–4	CC^Maj^ ≥ 2	

CO^Maj^, IR^Maj^, CR^Maj^, and CC^Maj^: correspond to the second highest (for studies with 3 or 4 animals) or the third highest (for studies with 5 or 6 animals) of the mean CO, IR, CR, and CC scores (of gradings at 24, 48, and 72 h). UN GHS criteria (UN 2013) and the ECHA Guidance on the application of the CLP criteria for classification of studies with more than three animals (ECHA, 2012; chapter 3.3.2.3.2.2 In vivo data) were applied (i.e. 2 out of 3, 3 out of 4, 3 out of 5, or 4 out of six animals with a mean score above a classification cut-off)

*CO* corneal opacity, *IR* iritis, *CR* conjunctiva redness, *CC* conjunctiva chemosis

^a^Mean scores are calculated from gradings at 24, 48, and 72 h after instillation of the test material and used to determine the classification of the chemical based on CO^Maj^, IR^Maj^, CR^Maj^, and CC^Maj^

^b^All effects have to fully reverse within an observation period of normally 21 days. UN GHS provides the option to distinguish this single hazard category into two optional subcategories (not implemented in EU CLP): “Category 2A” (irritant to eyes) when any of the eye effects in any animal is not fully reversible within 7 days of observation (i.e. CO, IR, CR and/or CC > 0 at 7 ≤ day < 21); “Category 2B” (mildly irritant to eyes) when all observed eye effects are fully reversible within 7 days of observation (i.e. CO, IR, CR and CC = 0 at day 7 and beyond)

^c^Cat 1 also applies when CO = 4 or other severe reactions (e.g. destruction of cornea, discoloration of the cornea by a dye substance, adhesion, pannus, or interference with the function of the iris or other effects that impair sight) are observed at any time point in any rabbit during the observation period, and/or when effects that are not expected to reverse, or have not fully reversed within an observation period of normally 21 days (persistent effects) are observed (i.e. score > 0 on day 21 on any tissue in any animal)

### Within-test variability

To assess the within-test variability, the individual rabbit data were used. As mentioned above, mean CO, IR, CR, and CC values over the reading times at 24, 48, and 72 h were calculated for each animal. According to the Draize scale, the CO and CC grading can assume values of 0, 1, 2, 3, or 4. This means that for the individual rabbits, the mean over the reading times at 24, 48, and 72 h can take 13 different values (from 0 to 4 in steps of 0.33). This rule also applies to the other endpoints except that the maximum scores for IR and CR are 2 and 3, thus resulting in seven and ten possible mean values, respectively. Sometimes, the CO, IR, CR, or CC mean scores for the animals, and as a consequence, the second (*n* = 3 or 4) or third (*n* = 5 or 6) highest mean scores (CO^Maj^, IR^Maj^, CR^Maj^ or CC^Maj^), deviated from the possible values. Therefore, the CO^Maj^, IR^Maj^, CR^Maj^ and CC^Maj^ values were assigned to intervals ([0, 0.33[, [0.33, 0.67[, [0.67, 1[, [1, 1.33[, [1.33, 1.67[, [1.67, 2[, [2, 2.33[, [2.33, 2.67[, [2.67, 3[, [3, 3.33[, [3.33, 3.67[, [3.67, 4[, [4]) that were represented by the corresponding values of 0, 0.33, 0.67, 1, 1.33, 1.67, 2, 2.33, 2.67, 3, 3.33, 3.67, 4, respectively. The within-test variability of the individual mean tissue scores is presented by means of frequency tables (one for each tissue), with the individual rabbit mean CO, IR, CR, or CC scores of each study/chemical (rows) being presented against the study/chemical CO^Maj^, IR^Maj^, CR^Maj^ or CC^Maj^ values (columns), respectively. In this analysis, the chemicals were grouped according to their CO^Maj^, IR^Maj^, CR^Maj^ or CC^Maj^ value for each of the tissues evaluated.

The within-test variability of the individual mean tissue scores was also assessed by means of boxplots representing the minimum, first quartile, median, third quartile, and maximum. In this analysis, the chemicals were grouped according to their UN GHS/EU CLP classification and classification driver, e.g. the rabbits of all chemicals that were classified based on persistence only were pooled together (De Wever et al. 2012; Barroso et al. 2013).

### Resampling probabilities

In order to determine the effect of the within-test variability on the accuracy of the Draize eye test (in terms of misclassification), resampling was performed. The resampling probabilities were estimated based on the same set of individual data that were used to assess the within-test variability. First, the data set was grouped according to UN GHS/EU CLP classification and further subdivided by classification driver. In this way, it was assured that the rabbits used in the various resamplings always came from studies with chemicals classified with the same UN GHS/EU CLP category (i.e. No Cat, Cat 2, or Cat 1) and in some cases even only from studies with chemicals that were classified based on the same endpoint. Next, data on 10,000 simulated chemicals were generated, i.e. a random sample of three rabbits was drawn 10,000 times from the data pool without replacement (Rao 1966). This means that each animal entered a simulated chemical only once. Finally, the UN GHS/EU CLP classification criteria were applied for these simulated chemicals and predictive capacity (correct classification) was calculated by comparing the theoretical classification (resulting from the resampling approach) with the observed classification. All analyses were performed in R version 3.0.0.

## Results

### Overview of the compiled data set: distribution according to UN GHS/EU CLP classification

Table 1 shows the total number of in vivo Draize eye studies present in each of the databases used in the present analyses. The RCD (ECETOC, ZEBET, and LNS) contains 344 studies, of which 70 were excluded from the analyses for not fulfilling the quality criteria defined in chapter 2.1. The NCD in turn contains 2319 individual studies, of which 459 were excluded. In total, 274 RCD studies and 1,860 NCD studies were used in the present analyses. Table 1 also presents the distribution of UN GHS/EU CLP categories in the RCD and NCD. The proportion of classified chemicals in the RCD is 39.8 % (17.2 % Cat 2 and 22.6 % Cat 1), as compared to only 17.4 % (10.4 % Cat 2 and 6.9 % Cat 1) in the NCD. The RCD in general also contains more Cat 1 than Cat 2 chemicals, whereas the opposite is observed for the NCD (Table 1). The distribution of UN GHS/EU CLP categories observed in the NCD is expected to represent more closely the prevalence of Cat 2 and Cat 1 in the chemical world than what is observed in the RCD, since the NCD contains all chemicals registered by multiple industry sectors since 1981, while the RCD contains a limited number of chemicals that were put together in databases mainly to support validation studies.

### Effects driving UN GHS/EU CLP classification

The proportions of the in vivo studies that led to an UN GHS/EU CLP Cat 1 or Cat 2 classification based on a specific endpoint or on a combination of endpoints [here named classification drivers, as described by De Wever et al. (2012) and Barroso et al. (2013)] are presented in Tables 3 and 5 for Cat 1 and Cat 2, respectively.

**Table 3:** Proportion of Cat 1 chemicals that were classified according to specific classification drivers

Effects	Severity % (*n*)			Persistence only % (*n*)	
	CO^Maj a^ ≥ 3	CO = 4^b^ (and CO^Maj a^ < 3)	IR^Maj a^ > 1.5 only	CO	No CO
RCD (*n* = 62)	32.3 (20)	29.0 (18)	3.2 (2)	25.8 (16)	9.7 (6)
Reversible	3.2 (2)	1.6 (1)	0.0 (0)		
Persistent	11.3 (7)	14.5 (9)	0.0 (0)		
Unknown	17.7 (11)	12.9 (8)	3.2 (2)		
NCD (*n* = 129)	24.0 (31)	7.0 (9)	3.9 (5)	52.7 (68)	12.4 (16)
Reversible	0.8 (1)	0.8 (1)	0.0 (0)		
Persistent	8.5 (11)	6.2 (8)	2.3 (3)		
Unknown	14.7 (19)	0.0 (0)	1.6 (2)		

*CO* corneal opacity, *IR* iritis, *RCD* Reference Chemicals Databases, *NCD* European New Chemicals Database

^a^second (*n* = 3 or 4) or third (*n* = 5 or 6) highest mean score

^b^Observed any time during the observation period (before day 21)

The chemicals classified as Cat 1 in the RCD and NCD were subgrouped according to four main classification drivers (Table 3). All chemicals with the second (*n* = 3 or 4) or third (*n* = 5 or 6) highest animal mean CO score equal to or greater than 3 (CO^Maj^ ≥ 3) were grouped together. Chemicals classified as Cat 1 based on a CO equal to 4 observed any time during the observation period (before day 21) but having the second (*n* = 3 or 4) or third (*n* = 5 or 6) highest animal mean CO score less than 3 (CO = 4, and CO^Maj^ < 3) were pooled in a second group. The chemicals classified as Cat 1 based only on a second (*n* = 3 or 4) or third (*n* = 5 or 6) highest animal mean IR score greater than 1.5 (IR^Maj^ > 1.5 only) were placed in a third group. These first three groups represent classification drivers related to severity of ocular lesions. Finally, chemicals that did not show sufficiently severe ocular lesions (CO^Maj^ < 3, IR^Maj^ ≤ 1.5 and CO ≠ 4) but that were classified Cat 1 based on persistence of effects (Persistence only) were separated and further subdivided into those that showed persistence of CO and those that showed persistence of CR, CC and/or IR, but not CO (No CO).

The majority of the chemicals classified based on severity in both the RCD and NCD have CO^Maj^ ≥ 3 (RCD: 32.3 %; NCD: 24.0 %). In the RCD, also a large percentage of the chemicals showed CO = 4 (and CO^Maj^ < 3) (29.0 %). In both the RCD and NCD, Cat 1 classification based on IR^Maj^ > 1.5 only was quite rare (<4 %). In total, more Cat 1 chemicals were classified based on severity in the RCD (64.5 %) than in the NCD (34.9 %). On the other hand, the proportion of chemicals that were classified Cat 1 based on persistence only was 65.1 % in the NCD (52.7 % with CO persistence and 12.4 % with only CR, CC and/or IR persistence) and 35.5 % in the RCD (25.8 % with CO persistence and 9.7 % with only CR, CC and/or IR persistence). All of the 10–12 % Cat 1 chemicals that were classified based on persistence of effects other than corneal opacity (“No CO” in Table 3) always showed CR and/or CC persistent effects, and the grades were generally low (mostly ≤2 and many with a grade of 1). Only a few of these chemicals showed IR persistent effects (27 %, 6/22). Overall, 61.3 % of the Cat 1 chemicals from the RCD generated persistent effects in the rabbits in comparison with 82.1 % for the NCD. It should, however, be pointed out that for 33.8 and 16.3 % of the Cat 1 chemicals in the RCD and in the NCD, respectively, the study was stopped before day 21 and, as such, it is unknown if the effect(s) would have persisted until day 21. For two data sources of the RCD (ECETOC and ZEBET), individual data were available until day 21. For the third RCD data source (LNS), individual data were available only until day 14, together with comments indicating if effects were persisting at day 21. In the NCD, individual animal data were only available for the mean scores of days 1–3, again with only summary information being provided regarding the persistence of effects at day 21 (only the maximum score at the end of the observation period for the whole study is provided). In ECETOC and ZEBET, if an effect was persistent at day 21, this was mainly observed on the cornea (44.4 % of animals) followed by conjunctiva redness (34.7 %), conjunctiva chemosis (20.2 %) (39 % with persistent conjunctiva redness and/or chemosis) and iris (16.2 %) (Table 4). The severity of CO (grade 1, 2, 3, or 4) and IR (grade 1 or 2) on day 21 is more or less equally distributed over the grades, whereas for the conjunctiva effects, lower grades were observed more frequently.

**Table 4:** Distribution of the individual tissue scores on day 21 by endpoint for the chemicals from the RCD (ECETOC and ZEBET) that resulted in a persistent effect in at least one animal (*n* = 124 animals) ^a^

Endpoint	Tissue score (proportion of animals)				
	0	1	2	3	4
CO	54.0	13.7	12.1	8.1	10.5
IR	82.3	8.1	8.1	NA	NA
CR	63.7	24.2	8.9	1.6	NA
CC	78.2	11.3	7.3	1.6	0.0

*CO* corneal opacity, *IR* iritis, *CR* conjunctiva redness, *CC* conjunctiva chemosis, *NA* not applicable

^a^Individual data for day 21 were only available for ECETOC and ZEBET databases. For 2 out of 124 animals, the study stopped before day 21 and the tissue score was not reversed to 0; these two animals were therefore not included in this table

The chemicals classified as Cat 2 in the RCD and NCD were themselves subgrouped according to three main classification drivers (Table 5). All chemicals with the second (*n* = 3 or 4) or third (*n* = 5 or 6) highest animal mean CO score equal to or greater than 1 but less than 3 (1 ≤ CO^Maj^ < 3) were grouped together. The chemicals having the largest of the second (*n* = 3 or 4) or third (*n* = 5 or 6) highest animal mean CR and CC scores equal to or greater than 2 but having the second (*n* = 3 or 4) or third (*n* = 5 or 6) highest animal mean CO score less than 1 (CR^Maj^/CC^Maj^ ≥ 2 and CO^Maj^ < 1) were pooled into another group. Finally, the chemicals classified as Cat 2 based only on a second (*n* = 3 or 4) or third (*n* = 5 or 6) highest animal mean IR score equal to or greater than 1 but less than or equal to 1.5 (1 ≤ IR^Maj^ ≤ 1.5 only) were placed together in the last group.

**Table 5:** Proportion of Cat 2 chemicals that were classified according to specific classification drivers

Effects	Severity % (*n*)		
	1 ≤ CO^Maj a^ < 3	CR^Maj^/CC^Maj b^ ≥ 2 (and CO^Maj a^ < 1)	1 ≤ IR^Maj a^ ≤ 1.5 only
RCD (*n* = 47)	74.5 (35)	25.5 (12)	0.0 (0)
CR^Maj^ + CC^Maj^	36.2 (17)	6.4 (3)	NA
CR^Maj^ only	21.3 (10)	17.0 (8)	NA
CC^Maj^ only	6.4 (3)	2.1 (1)	NA
CO^Maj^ only	10.6 (5)	NA	NA
NCD (*n* = 194)	54.1 (105)	43.3 (84)	2.6 (5)
CR^Maj^ + CC^Maj^	14.9 (29)	7.2 (14)	NA
CR^Maj^ only	18.6 (36)	34.0 (66)	NA
CC^Maj^ only	0.5 (1)	2.1 (4)	NA
CO^Maj^ only	20.1 (39)	NA	NA

*CO* corneal opacity, *IR* iritis, *CR* conjunctiva redness, *CC* conjunctiva chemosis, *RCD* Reference Chemicals Databases, *NCD* European New Chemicals Database, *NA* not applicable

^a^second (*n* = 3 or 4) or third (*n* = 5 or 6) highest mean score

^b^The largest of the second (*n* = 3 or 4) or third (*n* = 5 or 6) highest mean CR and CC scores

The majority of the Cat 2 chemicals showed sufficient CO effects to generate a Cat 2 classification per se (RCD: 74.5 %; NCD: 54.1 %), but these were often accompanied by conjunctiva effects that would also trigger a Cat 2 classification (86 and 63 % of the RCD and NCD Cat 2 chemicals classified based on CO effects, respectively, representing 63.9 and 34.0 % of all Cat 2 chemicals in the RCD and NCD, respectively) (Table 5). Importantly, also a considerable amount of Cat 2 chemicals were classified based on conjunctiva effects without showing classifiable CO effects (RCD: 25.5 %; NCD: 43.3 %). Altogether, an impressive 89.4 and 77.3 % of the Cat 2 chemicals in the RCD and NCD, respectively, showed sufficient conjunctiva effects to generate a Cat 2 classification per se. Of the two conjunctiva effects, CR is the predominant one in all databases, with CC rarely appearing without parallel CR effects (<10 %). A very small proportion (2.6 %) of the chemicals in the NCD was classified Cat 2 because of iritis only. The effects on the iris seem therefore to be of lesser importance for classification of chemicals under the UN GHS/EU CLP system.

Another important observation in the RCD data set is that high CO scores (≥3) were able to reverse to 0 by day 21 or earlier. Of the 66 animals with CO = 3 scored any time during the observation period (before day 21), 33 (50 %) recovered to CO = 0 by day 21 or earlier. CO > 0 at day 21 was observed for 15 (22.7 %) animals, and no conclusion could be drawn for 18 (27.3 %) animals because the study was stopped before day 21. Of note, even 8.8 % (5/57) of the animals with extreme CO scores (CO = 4) noted any time during the observation period (before day 21) recovered to CO = 0 by day 21 or earlier, whereas CO persistence at day 21 was observed in 29.8 % (17/57) of these animals. As expected, the study was stopped before day 21 for a large proportion of these animals (61.4 %, 35/57), due to animal welfare concerns, and therefore, it remains unknown if full recovery could occur in any of these cases. Interestingly, mean CO scores over the reading times at 24, 48, and 72 h ≥3, reversed to 0 by day 21 or earlier in as high as 19.3 % (11/57) of the animals.

### Within-test variability of tissue scores

Mean tissue scores (calculated over the reading times at 24, 48, and 72 h for an individual animal) were available for all rabbits tested with the RCD and NCD chemicals included in the analyses presented in this study. These data were used to assess the within-test variability of each individual tissue scoring (Tables 6, 7, 8, 9).

**Table 6:**
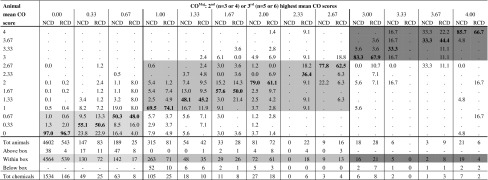
Within-test variability of the mean CO scores calculated over the reading times at 24, 48, and 72 h. Proportion of animals within each CO^Maj^ group

Proportions that fall in the light-grey area (No Cat range) correspond to animals with mean CO < 1 that were tested with chemicals with CO^Maj^ < 1; Proportions that fall in the grey area (Cat 2 range) correspond to animals with 1 ≤ mean CO < 3 that were tested with chemicals with 1 ≤ CO^Maj^ < 3; Proportions that fall in the dark-grey area (Cat 1 range) correspond to animals with mean CO ≥ 3 that were tested with chemicals with CO^Maj^ ≥ 3; Proportions in bold (diagonal) correspond to animals with mean CO scores equal to the CO^Maj^ of the chemicals tested in those animals

*CO* corneal opacity,* NCD* European New Chemicals Database, *RCD* Reference Chemicals Databases

**Table 7:**
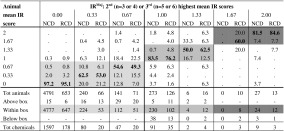
Within-test variability of the mean IR scores calculated over the reading times at 24, 48, and 72 h. Proportion of animals within each IR^Maj^ group

Proportions that fall in the light-grey area (No Cat range) correspond to animals with mean IR < 1 that were tested with chemicals with IR^Maj^ < 1; Proportions that fall in the grey area (Cat 2 range) correspond to animals with 1 ≤ mean IR ≤ 1.5 that were tested with chemicals with 1 ≤ IR^Maj^ ≤ 1.5; Proportions that fall in the dark-grey area (Cat 1 range) correspond to animals with mean IR > 1.5 that were tested with chemicals with IR^Maj^ > 1.5; Proportions in bold (diagonal) correspond to animals with mean CO scores equal to the IR^Maj^ of the chemicals tested in those animals

*IR* iritis, *NCD* European New Chemicals Database, *RCD* Reference Chemicals Databases

**Table 8:**
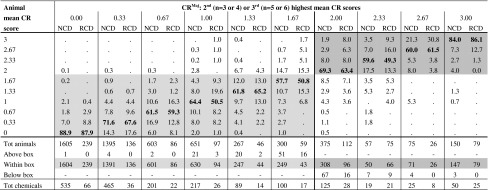
Within-test variability of the mean CR scores calculated over the reading times at 24, 48, and 72 h. Proportion of animals within each CR^Maj^ group

Proportions that fall in the light-grey area (No Cat range) correspond to animals with mean CR < 2 that were tested with chemicals with CR^Maj^ < 2; Proportions that fall in the grey area (Cat 2 range) correspond to animals with mean CR ≥ 2 that were tested with chemicals with CR^Maj^ ≥ 2; Proportions in bold (diagonal) correspond to animals with mean CR scores equal to the CR^Maj^ of the chemicals tested in those animals

*CR* conjunctiva redness, *NCD* European New Chemicals Database, *RCD* Reference Chemicals Databases

**Table 9:**
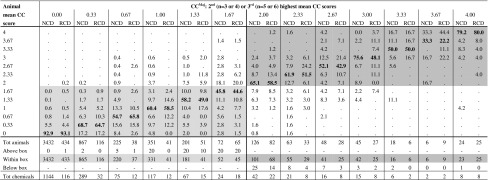
Within-test variability of the mean CC scores calculated over the reading times at 24, 48, and 72 h. Proportion of animals within each CC^Maj^ group

Proportions that fall in the light-grey area (No Cat range) correspond to animals with mean CC < 2 that were tested with chemicals with CC^Maj^ < 2; Proportions that fall in the grey area (Cat 2 range) correspond to animals with mean CC ≥ 2 that were tested with chemicals with CC^Maj^ ≥ 2; Proportions in bold (diagonal) correspond to animals with mean CC scores equal to the CC^Maj^ of the chemicals tested in those animals

*CC* conjunctiva chemosis, *NCD* European New Chemicals Database, *RCD* Reference Chemicals Databases

According to the Draize eye test scoring system, the mean CO calculated over the reading times at 24, 48, and 72 h of an individual animal can take 13 possible values, which are shown as individual rows in Table 6. Consequently, the CO^Maj^ of each individual study/chemical can only assume one of those 13 values (see chapter 2.4 in the “Materials and Methods”), which are shown as individual columns in Table 6. A chemical tested in three animals with mean animal CO scores of 0, 0.33, 0.33 (second highest value = 0.33) places the chemical in the column corresponding to CO^Maj^ equal to 0.33, with the individual animal mean scores added in the respective rows (i.e. 2 counted in the row corresponding to 0.33, and 1 counted in the row corresponding to 0). In total, 25 chemicals (83 animals) of the RCD had CO^Maj^ score equal to 0.33, with individual mean CO scores ranging from 0 (22.9 % of the animals) to 2.67 (1.2 % of the animals).

The within-test variability for the mean CO, IR, CR, and CC scores was very similar for the RCD and NCD (Tables 6, 7, 8, 9). These figures only reflect the variability observed over the first 3 days for each tissue effect separately and do not take into account the timing of reversibility. Overall, only a small proportion of the animals from chemicals with CO^Maj^, IR^Maj^, CR^Maj^ or CC^Maj^ scores below the Cat 2 classification threshold (CO^Maj^ < 1; IR^Maj^ < 1; CR^Maj^ < 2; CC^Maj^ < 2) had individual mean tissue scores above the Cat 2 classification threshold for the respective tissue: 3.5 % for CO (23/651), 4.9 % for IR (39/790), 3.2 % for CR (21/663) and 4.3 % for CC (32/745) of the animals in the RCD and 2.1 % for CO (102/4,938), 1.2 % for IR (60/5,172), 2.1 % for CR (99/4,821), and 1.3 % for CC (67/5,148) of the animals in the NCD (all animals counted above the light-grey boxes in Tables 6, 7, 8, and 9). A higher variability was observed for chemicals with CO^Maj^ or IR^Maj^ between the Cat 2 and the Cat 1 classification thresholds (1 ≤ CO^Maj^ < 3; 1 ≤ IR^Maj^ ≤ 1.5) or with CR^Maj^ or CC^Maj^ above the Cat 2 classification threshold (CR^Maj^ ≥ 2; CC^Maj^ ≥ 2). For 14.2 % (37/261), 19.7 % (28/142), 8.6 % (25/292), and 11 % (23/210) of the animals from the RCD and 14.4 % (71/492), 16.1 % (45/279), 12.3 % (81/657), and 13.9 % (46/330) of the animals from the NCD the mean tissue scores were outside the region delimited by the Cat 2 and Cat 1 classification thresholds for CO (mean CO < 1 or mean CO ≥ 3) and IR (mean IR < 1 or mean IR > 1.5) (all animals counted above and below the grey boxes in Table 6 and 7), and below the Cat 2 classification threshold for CR and CC (mean CR < 2; mean CC < 2) (all animals counted below the grey boxes in Tables 8 and 9), respectively. Moreover, for the chemicals with CO^Maj^ between the Cat 2 and the Cat 1 classification thresholds (1 ≤ CO^Maj^ < 3), 7.7 % (20/261) of the animals from the RCD had mean CO scores below the Cat 2 classification threshold (mean CO < 1) and 6.5 % (17/261) had mean CO scores above the Cat 1 classification threshold (mean CO ≥ 3). In the NCD, on the other hand, 13.2 % (65/492) of the animals had mean CO scores below the Cat 2 classification threshold (mean CO < 1) and only 1.2 % (6/492) of the animals had mean CO scores above the Cat 1 classification threshold (mean CO ≥ 3). Similarly, for the chemicals with IR^Maj^ between the Cat 2 and the Cat 1 classification thresholds (1 ≤ IR^Maj^ ≤ 1.5), 10.6 % (15/142) of the animals from the RCD had mean IR scores below the Cat 2 classification threshold (mean IR < 1) and 9.2 % (13/142) had mean IR scores above the Cat 1 classification threshold (mean IR > 1.5), while in the NCD, 13.6 % (38/279) of the animals had mean IR scores below the Cat 2 classification threshold (mean IR < 1), and only 2.5 % (7/279) of the animals had mean IR scores above the Cat 1 classification threshold (mean IR > 1.5). Finally, for the chemicals with CO^Maj^ or IR^Maj^ above the Cat 1 classification threshold (CO^Maj^ ≥ 3; IR^Maj^ > 1.5), a substantial 23.3 % (10/43) or 13.0 % (3/23) (RCD) and 12.5 % (6/48) or 11.1 % (3/27) (NCD) of the animals had mean CO or IR scores, respectively, below the Cat 1 classification threshold (all animals counted below the dark-grey box in Tables 6 and 7).

### Within-test variability of mean tissue scores per classification driver

**Fig. 1:**
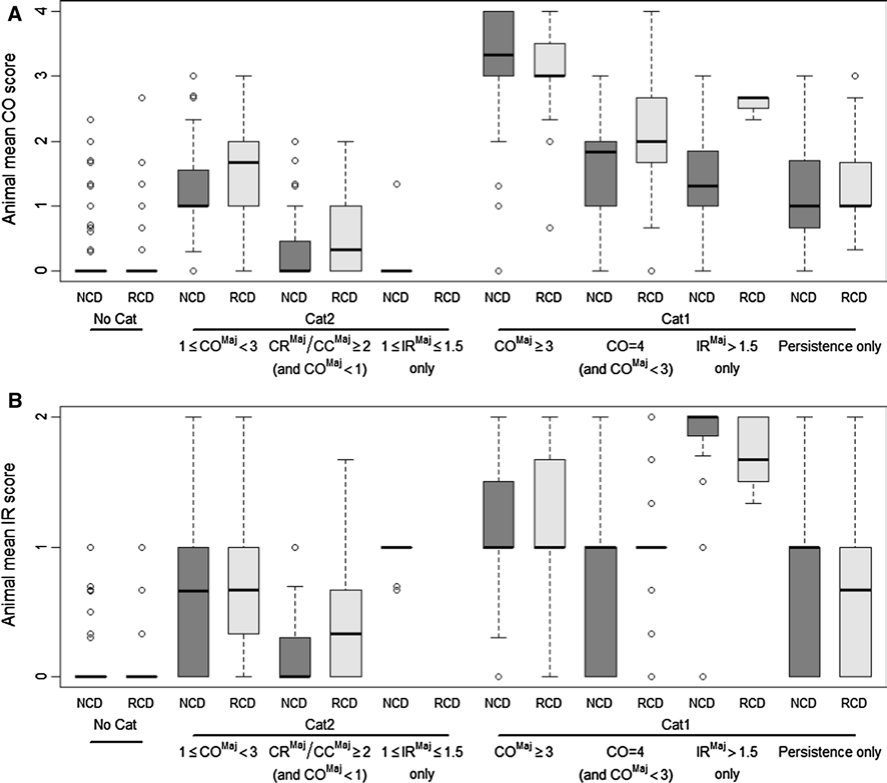
Boxplots presenting the distribution of individual animal mean CO (**a**) and mean IR (**b**) scores calculated over the reading times at 24, 48, and 72 h by classification driver. NCD: European New Chemicals Database, RCD: Reference Chemicals Databases, No Cat: not classified, Cat 2—1 ≤ CO^Maj^ < 3: classified based on majority of mean CO scores equal to or greater than 1 but less than 3, Cat 2—CR^Maj^/CC^Maj^ ≥ 2 (and CO^Maj^ < 1): classified based on majority of mean CR and/or CC scores equal to or greater than 2 but with majority of mean CO scores less than 1, Cat 2—1 ≤ IR^Maj^ ≤ 1.5 only: classified based on majority of mean IR scores equal to or greater than 1 but less than or equal to 1.5, Cat 1—CO^Maj^ ≥ 3: classified based on majority of mean CO scores equal to or greater than 3, Cat 1—CO = 4 (and CO^Maj^ < 3): classified based on CO = 4 but with majority of mean CO scores less than 3, Cat 1—IR^Maj^ > 1.5 only: classified based on majority of mean IR scores greater than 1.5, Cat 1—Persistence only: classified based on persistence only. The *whiskers* correspond with the smallest and largest observation that fall within a distance of 1.5 times the length of the *box* (Interquartile Range, IQR) from the lower (*bottom side* of the *box*) and upper quartile (*upper side* of the *box*), respectively

**Fig. 2:**
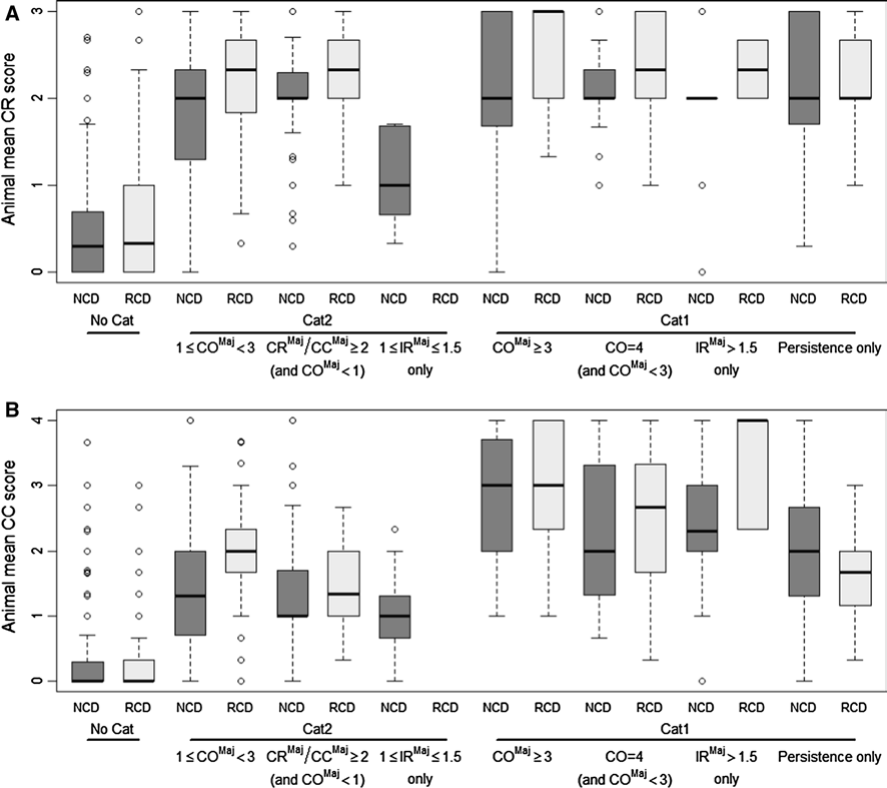
Boxplots presenting the distribution of individual animal mean CR (**a**) and mean CC (**b**) scores calculated over the reading times at 24, 48, and 72 h by classification driver. NCD: European New Chemicals Database, RCD: Reference Chemicals Databases, No Cat: not classified, Cat 2—1 ≤ CO^Maj^ < 3: classified based on majority of mean CO scores equal to or greater than 1 but less than 3, Cat 2—CR^Maj^/CC^Maj^ ≥ 2 (and CO^Maj^ < 1): classified based on majority of mean CR and/or CC scores equal to or greater than 2 but with majority of mean CO scores less than 1, Cat 2—1 ≤ IR^Maj^ ≤ 1.5 only: classified based on majority of mean IR scores equal to or greater than 1 but less than or equal to 1.5, Cat 1—CO^Maj^ ≥ 3: classified based on majority of mean CO scores equal to or greater than 3, Cat 1—CO = 4 (and CO^Maj^ < 3): classified based on CO = 4 but with majority of mean CO scores less than 3, Cat 1—IR^Maj^ > 1.5 only: classified based on majority of mean IR scores greater than 1.5, Cat 1—Persistence only: classified based on persistence only. The *whiskers* correspond with the smallest and largest observation that fall within a distance of 1.5 times the length of the *box* (Interquartile Range, IQR) from the lower (*bottom side* of the *box*) and upper quartile (*upper side* of the *box*), respectively

The within-test variability of the mean tissue scores was further evaluated by grouping the data of the different studies available in the RCD and NCD according to the UN GHS/EU CLP classification of the tested chemicals and their classification driver as described above (De Wever et al. 2012; Barroso et al. 2013). These data are presented in boxplots that illustrate the distribution of the animals mean CO score (Fig. 1a), mean IR score (Fig. 1b), mean CR score (Fig. 2a), and mean CC score (Fig. 2b), per classification driver.

Chemicals not classified for serious eye damage/eye irritation (No Cat) in both the RCD and NCD have in the majority of the animals mean CO scores and mean IR scores equal to 0 (Fig. 1). The majority of these animals also have mean CC scores less than or equal to 0.33 (Fig. 2b). Of note, the mean CR scores are equal to or greater than 0.33 but less than 2 for the majority of these animals in both databases (Fig. 2a).

In both the RCD and NCD, the majority of the animals tested with chemicals that were classified Cat 2 based on 1 ≤ CO^Maj^ < 3 have mean CO scores between the Cat 2 and the Cat 1 classification thresholds (Fig. 1a). However, the animal mean CO scores are generally lower in the NCD than in the RCD. The mean IR scores in the NCD and RCD, on the other hand, are less than 1 in the majority of the animals tested with this group of chemicals (Fig. 1b). Furthermore, in both the NCD and RCD, the mean CR scores of the Cat 2 chemicals that were classified based on 1 ≤ CO^Maj^ < 3 are equal to or greater than 2 in the majority of the animals (75.0 % (93/124) in the RCD and 59.4 % (187/315) in the NCD), but with a larger proportion being below this score in the NCD than in the RCD (Fig. 2a). The same was observed for the animal mean CC scores in the RCD database. For the NCD, however, the mean CC scores are less than 2 in the majority of the animals. In the NCD and RCD, the majority of the animals tested with chemicals that were classified Cat 2 based on conjunctiva effects, but without enough corneal involvement to generate a classification, have mean CR scores that are above the Cat 2 classification threshold (Fig. 2a) but have mean CO, IR, and CC scores that are below the Cat 2 classification threshold (Figs. 1a, 2a, b). This means that the great majority of these chemicals were classified based on CR^Maj^ only (see also Table 5).

In both the RCD and NCD, the majority of the animals tested with chemicals that were classified Cat 1 based on CO^Maj^ ≥ 3 have mean CO scores equal to or greater than 3 (Fig. 1a). The mean CO scores of the animals tested with chemicals that were classified Cat 1 based on a CO = 4 (and CO^Maj^ < 3), IR^Maj^ > 1.5 only or Persistence only are generally above the CO Cat 2 classification threshold and mostly below the Cat 1 classification threshold (1 ≤ mean CO < 3) for both databases (Fig. 1a). It is important to note, however, that for the NCD, about 25 % (50/198) of the animals tested with chemicals that were classified Cat 1 based on persistence only have both mean CO and mean IR scores less than 1 (Fig. 1). The mean CR scores of the animals tested with classified chemicals cover in general the same range independently of the classification (Cat 2 or Cat 1) or the classification driver (Fig. 2a), which means that practically no distinction can be made between Cat 2 and Cat 1 chemicals on the basis of CR. On the other hand, the mean CC scores of especially the Cat 1 chemicals that were classified based on CO^Maj^ (CO^Maj^ ≥ 3), CO = 4 (and CO^Maj^ < 3), or IR^Maj^ (IR^Maj^ > 1.5) are generally higher than the mean CC scores of the Cat 2 chemicals (Fig. 2b).

### Resampling probabilities

The influence of within-test variability on the UN GHS/EU CLP chemical classification was further explored using a statistical resampling technique without replacement. Only studies with individual data on at least three rabbits were taken into account. In the resampling approach used in this study, simulated chemicals were created by randomly grouping together three animals that may have been tested with different chemicals (for details see chapter 2.5 in the “Materials and Methods”). For this reason, the different studies available in the RCD and NCD were first pooled according to the UN GHS/EU CLP classification of the tested chemicals and also further subdivided according to their classification driver as described above (De Wever et al. 2012; Barroso et al. 2013). Separate resampling analyses were then performed on each of these individual data pools. The individual simulated chemicals obtained from the different resamplings were therefore built either (1) on animals coming from studies with chemicals classified with the same UN GHS/EU CLP category (i.e. No Cat, Cat 2, or Cat 1) independently of the classification driver (taking all classification drivers together) or (2) on animals coming from studies with chemicals classified with the same UN GHS/EU CLP category and having the same endpoint driving such classification. For the chemicals of the NCD, only the individual animal mean tissue scores calculated over the reading times at 24, 48, and 72 h are available. Information on persistence of effects is only available in the form of comments, often not specifying in which tissues and in how many animals the persistence of effects was observed. It was therefore not possible to estimate the resampling probabilities for the chemicals that were classified based only on CO = 4 (observed any time during the observation period, before day 21) or persistence of a tissue effect.

In the RCD, 165 chemicals were not classified for serious eye damage/eye irritation according to the UN GHS/EU CLP classification criteria (No Cat). This group consisted of 606 individual animals (Table 10). A random sample of three rabbits was drawn 10,000 times from this data pool of 606 rabbits, and the UN GHS/EU CLP classification criteria were applied. As can be seen in Table 10, almost all of the simulated chemicals were also identified as No Cat (99.9 %). Only 0.1 % (8 chemicals) were classified Cat 2, and none of the simulated chemicals were classified Cat 1. Identical results were obtained for the NCD data pool. Thus, resampling from the RCD and NCD data pools of the non-classified compounds rarely resulted in misclassifications, which may be explained by the fact that the great majority of the individual animal mean CO, IR, CR, and CC scores observed for the No Cat chemicals in both databases are below the Cat 2 classification cut-offs (Figs. 1, 2).

**Table 10:** Resampling probabilities of 10,000 theoretical chemicals according to UN GHS/EU CLP criteria

True class	Database	Endpoint	Sample size	Predicted class		
				No Cat	Cat 2	Cat 1
No Cat	RCD		606	**99.9**	0.1	0.0
	NCD		4,611	**99.9**	0.1	0.0
Cat 2	RCD	All^a^	162	3.9	**95.5**	0.6
		1 ≤ CO^Maj b^ < 3	124	2.3	**97.2**	0.6
		CR^Maj^/CC^Maj c^ ≥ 2 (and CO^Maj b^ < 1)	38	6.0	**94.1**	NA
	NCD	All	582	11.7	**88.2**	0.1
		1 ≤ CO^Maj b^ < 3	315	5.0	**94.8**	0.2
		CR^Maj^/CC^Maj c^ ≥ 2 (and CO^Maj b^ < 1)	252	9.7	**90.3**	NA
		1 ≤ IR^Maj b^ ≤ 1.5 only	15	7.6	**92.4**	0.0
Cat 1	RCD	All	187	0.0	10.8	**89.2**
		CO^Maj b^ ≥ 3	43	0.0	1.7	**98.3**
		CO = 4^d^ (and CO^Maj b^ < 3)	61	0.0	13.0	**87.0**
		IR^Maj b^ > 1.5 only	7	0.0	14.2	**85.8**
		Persistence only	76	0.1	13.5	**86.4**
	NCD	CO^Maj b^ ≥ 3 + IR^Maj b^ > 1.5 only^e^	63	0.0	7.2	**92.8**
		CO^Maj b^ ≥ 3	48	0.0	1.9	**98.1**
		IR^Maj b^ > 1.5 only	15	0.0	8.2	**91.8**

Proportions in bold represent agreement between the predicted classification and the true classification

*RCD* Reference Chemicals Databases, *NCD* European New Chemicals Database, *CO* corneal opacity, *CR* conjunctiva redness, *CC* conjunctiva chemosis, *IR* iritis, *NA* not applicable

^a^None of the chemicals in this group was classified based on the driver 1 ≤ IR^Maj^ ≤ 1.5 only

^b^second (*n* = 3 or 4) or third (*n* = 5 or 6) highest mean score

^c^The largest of the second (*n* = 3 or 4) or third (*n* = 5 or 6) highest mean CR and CC scores

^d^Observed any time during the observation period (before day 21)

^e^Individual animal data only accessible for the mean scores of days 1–3. Information on persistence of effects is only available in the form of comments, often not specifying in which tissues and in how many animals the persistence of effects was observed. Consequently, it was not possible to estimate the resampling probabilities for the chemicals that were classified based only on CO = 4 (observed any time during the observation period, before day 21) or persistence of a tissue effect. The resampling probabilities reported for the classification drivers CO^Maj^ ≥ 3 and IR^Maj^ > 1.5 should therefore not be considered as representative of the overall resampling probabilities for chemicals classified as Cat 1 in the NCD

The total number of animals available for estimating the resampling probabilities for the UN GHS/EU CLP Cat 2 was 162 for the RCD and 582 for the NCD (Table 10). Only a small proportion of the simulated chemicals from the RCD was under-predicted (3.9 %) and very few were over-predicted (0.6 %). For the NCD, however, the under-prediction rate was substantially higher (11.7 %) but again only 0.1 % of the simulated chemicals were predicted as Cat 1. Separate resamplings were also performed for each Cat 2 classification driver. For the Cat 2 chemicals that were classified based on CO^Maj^, the majority of the animals in both the RCD and NCD have mean CO scores between the Cat 2 and the Cat 1 classification thresholds (1 ≤ mean CO < 3) (Fig. 1a). Moreover, many of the animals in the 1 ≤ CO^Maj^ < 3 RCD and NCD data pools also showed mean CR scores equal to or greater than 2 (Fig. 2a; Table 5). This resulted in a low resampling misclassification error for these data pools with more than 94 % of the simulated chemicals being correctly predicted as Cat 2 eye irritants (Table 10). A higher misclassification error was observed for the chemicals classified as Cat 2 based on conjunctiva effects and without enough corneal involvement to generate a classification (CR^Maj^/CC^Maj^ ≥ 2 and CO^Maj^ < 1), with under-prediction rates of 6.0 and of 9.7 % being obtained for the RCD and NCD, respectively. The majority of these chemicals were classified based on CR^Maj^ (Table 5: only 2.1 % in both the RCD and NCD were classified based on CC^Maj^ only), but some of the individual animal mean CR scores were still below the Cat 2 classification threshold of 2, with the median of all values being at (NCD) or very close to (RCD) this cut-off score (Fig. 2a). More importantly, the great majority of the individual animal’ mean CC scores for these chemicals were below this threshold (Fig. 2b). In fact, altogether 17 and 13 % of the animals from chemicals in the NCD and RCD, respectively, that were classified as Cat 2 based on conjunctiva effects, but without enough corneal involvement to generate a classification, had mean CR and CC scores <2. This indeed accounts for the higher variability observed for these chemicals. Only the NCD contains chemicals classified as Cat 2 based on iritis only (1 ≤ IR^Maj^ ≤ 1.5 only). These showed an under-classification rate of 7.6 %, which is in between the 5.0 % obtained for CO^Maj^ and the 9.7 % obtained for CR^Maj^/CC^Maj^. Overall, the under-classification errors for the Cat 2 chemicals were always higher than the over-classification errors, with the latter being almost neglectable.

As explained above, resampling from the whole Cat 1 data pool could only be performed for the chemicals from the RCD. When all classification drivers were taken into account in the RCD, resampling resulted in an under-prediction rate as Cat 2 of 10.8 % and practically no under-prediction as NC (Table 10). Under-predictions as NC were actually only observed for a small proportion of the simulated chemicals that were classified based on persistence only (0.1 % or 13/10,000) (Table 10). The classification drivers that most contributed to the overall under-prediction rate observed for all Cat 1 RCD chemicals were persistence of effects and CO = 4, each showing individually an under-prediction rate of about 13 %. The chemicals classified as Cat 1 based only on persistence of effects have mean CO scores of similar grade as the Cat 2 chemicals that were classified based on CO^Maj^ ≥ 1 (Fig. 1a). However, in the NCD, more than 25 % of the animals from this group of Cat 1 chemicals have mean CO scores <1 (Fig. 1a), which strongly suggests that the under-prediction rate for this subgroup is even higher than what is observed in the RCD (>13.5 %). The mean CO scores for the chemicals classified as Cat 1 based on CO = 4, on the other hand, were clearly higher than those for the chemicals classified as Cat 2 based on CO^Maj^ ≥ 1, but the majority were still well within the Cat 2 scores, i.e. below the Cat 1 classification threshold (Fig. 1a). This may still explain the high variability observed for this classification driver. Interestingly, the mean CO scores for the chemicals classified as Cat 1 based on CO = 4 were markedly higher for the RCD than for the NCD (Fig. 1a), suggesting that the under-prediction rate of the NCD is probably also higher for these chemicals than that of the RCD (>13.0 %). In fact, the misclassification rates were always higher in the NCD than in the RCD for both Cat 1 and Cat 2 chemicals as well as for all individual classification drivers. It is therefore expected that the overall variability for Cat 1 in the NCD (if the chemicals classified based on CO = 4 only and on persistence of effects only could also be considered) is at least as high as the one obtained with the RCD and probably even higher (>10.8 %). A considerable under-prediction error of 14.2 % (RCD) and 8.2 % (NCD) was obtained for the chemicals classified as Cat 1 based on IR^Maj^ > 1.5 only. Finally, chemicals that were classified Cat 1 based on CO^Maj^ ≥ 3 showed a low under-prediction rate in both the RCD (1.7 %) and NCD (1.9 %) (Table 10). This result is expected considering that the CO^Maj^ ≥ 3 data pool encloses several Cat 1 classification drivers. All chemicals in this group meet the CO^Maj^ ≥ 3 criterion but some will also meet one or several of the other Cat 1 criteria as well, in particular persistence of effects and/or CO = 4. For example, 53.8 % (23/43) of the animals in the RCD CO^Maj^ ≥ 3 data pool also have a CO = 4 observed at least at one time point during the observation period. Moreover, the majority of the animals in this group have mean CO scores ≥3 (87.5 % in the NCD and 76.7 % in the RCD). This will of course decrease the variability of this data pool, since the probability of meeting at least one of the Cat 1 criteria in the resampled chemicals is quite high. Accordingly, of all the Cat 1 classification drivers, CO^Maj^ ≥ 3 was the one showing the lowest variability.

### Resampling probabilities in function of physical state

It was further explored if the physical state of the chemical had an impact on the within-test variability. Therefore, the same resampling analyses as described in the previous section were performed on the liquids and solids separately. These results are presented in Tables 11 and 12. Data on the physical state could not be retrieved for 20 chemicals from the ZEBET database (RCD) and for one chemical of the NCD, and therefore, these chemicals were not included in these analyses. The separate resampling for the liquids and solids of the NCD resulted in a slightly higher overall under-prediction of Cat 2 chemicals for the solids (12.7 %) than for the liquids (9.8 %). The misclassification error for the 1 ≤ CO^Maj^ < 3 classification driver was also higher for solids (5.6 %) than for liquids (3.5 %), but the opposite was observed for the CR^Maj^/CC^Maj^ ≥ 2 (without CO^Maj^ ≥ 1) classification driver, with under-prediction rates of 11.9 and 9.2 % being obtained for the Cat 2 liquids and solids, respectively. The exact same trends were observed for the Cat 2 chemicals from the RCD, but the difference between liquids and solids for the CR^Maj^/CC^Maj^ ≥ 2 (without CO^Maj^ ≥ 1) classification driver was substantially higher, with the liquids showing a much larger under-prediction rate (12.0 %) than the solids (1.3 %). Another important difference between the liquids and solids of the RCD was observed for the Cat 1 chemicals classified based only on persistence of effects, with the under-prediction rate as Cat 2 obtained for the liquids being almost 12 % higher than for the solids. The solids showed an already high under-prediction rate of 10.4 %, but the under-prediction rate obtained with the liquids was as large as 22.2 %. Not surprisingly, 7 out of the 10 liquids from this data pool showed persistence of effects in only one of the tested animals, with four of these being 6 animal studies (two times CO persistence and two times CR persistence only). As expected, this led to a very large variability upon resampling of the data, with even a few under-predictions as No Cat being obtained (0.1 %). In fact, persistence only for the RCD liquids was the only classification driver that showed under-prediction of Cat 1 chemicals as No Cat. No important difference was observed between liquids and solids for the remaining Cat 1 classification drivers that could be resampled separately for both physical states, i.e. CO^Maj^ ≥ 3 and CO = 4.

**Table 11:** Resampling probabilities of 10,000 theoretical chemicals according to UN GHS/EU CLP criteria*—*liquids

True class	Database	Endpoint	Sample size	Predicted class		
				No Cat	Cat 2	Cat 1
No Cat	RCD		399	**99.9**	0.1	0.0
	NCD		1,002	**99.9**	0.1	0.0
Cat 2	RCD	All^a^	123	3.2	**96.2**	0.6
		1 ≤ CO^Maj b^ < 3	103	2.1	**97.5**	0.5
		CR^Maj^/CC^Maj c^ ≥ 2 (and CO^Maj b^ < 1)	20	12.0	**88.0**	NA
	NCD	All^a^	156	9.8	**90.2**	0.0
		1 ≤ CO^Maj b^ < 3	99	3.5	**96.5**	0.0
		CR^Maj^/CC^Maj c^ ≥ 2 (and CO^Maj b^ < 1)	57	11.9	**88.1**	NA
Cat 1	RCD	All	94	0.0	15.3	**84.7**
		CO^Maj b^ ≥ 3	25	0.0	1.4	**98.6**
		CO = 4^d^ (and CO^Maj b^ < 3)	22	0.0	12.1	**87.9**
		IR^Maj b^ > 1.5 only	4^e^	NA	NA	NA
		Persistence only	43	0.1	22.2	**77.7**
	NCD	CO^Maj b^ ≥ 3 + IR^Maj b^ > 1.5 only^f^	9	1.1	1.3	**97.7**
		CO^Maj b^ ≥ 3	6	0.0	0.0	**100.0**
		IR^Maj b^ > 1.5 only	3^e^	NA	NA	NA

Proportions in bold represent agreement between the predicted classification and the true classification

*RCD* Reference Chemicals Databases, *NCD* European New Chemicals Database, *CO* corneal opacity, *CR* conjunctiva redness, *CC* conjunctiva chemosis, *IR* iritis, *NA* not applicable

^a^None of the chemicals in this group was classified based on the driver 1 ≤ IR^Maj^ ≤ 1.5 only

^b^second (*n* = 3 or 4) or third (*n* = 5 or 6) highest mean score

^c^The largest of the second (*n* = 3 or 4) or third (*n* = 5 or 6) highest mean CR and CC scores

^d^Observed any time during the observation period (before day 21)

^e^Sample size is too small to perform a separate resampling analysis for this driver

^f^Individual animal data only accessible for the mean scores of days 1–3. Information on persistence of effects is only available in the form of comments, often not specifying in which tissues and in how many animals the persistence of effects was observed. Consequently, it was not possible to estimate the resampling probabilities for the chemicals that were classified based only on CO = 4 (observed any time during the observation period, before day 21) or persistence of a tissue effect. The resampling probabilities reported for the classification drivers CO^Maj^ ≥ 3 and IR^Maj^ > 1.5 should therefore not be considered as representative of the overall resampling probabilities for chemicals classified as Cat 1 in the NCD

**Table 12:** Resampling probabilities of 10,000 theoretical chemicals according to UN GHS/EU CLP criteria*—*solids

True class	Database	Endpoint	Sample size	Predicted class		
				No Cat	Cat 2	Cat 1
No Cat	RCD		152	**100.0**	0.0	0.00
	NCD		3,606	**99.9**	0.1	0.00
Cat 2	RCD	All^a^	33	5.0	**95.0**	0.0
		1 ≤ CO^Maj b^ < 3	15	2.8	**97.2**	0.0
		CR^Maj^/CC^Maj c^ ≥ 2 (and CO^Maj b^ < 1)	18	1.3	**98.7**	NA
	NCD	All	426	12.7	**87.2**	0.0
		1 ≤ CO^Maj b^ < 3	216	5.6	**94.3**	0.1
		CR^Maj^/CC^Maj c^ ≥ 2 (and CO^Maj b^ < 1)	195	9.2	**90.8**	NA
		1 ≤ IR^Maj b^ ≤ 1.5 only	15	7.6	**92.4**	0.0
Cat 1	RCD	All	78	0.0	8.2	**91.9**
		CO^Maj b^ ≥ 3	18	0.0	1.1	**98.7**
		CO = 4^d^ (and CO^Maj b^ < 3)	39	0.0	12.7	**87.3**
		IR^Maj b^ > 1.5 only	3^e^	NA	NA	NA
		Persistence only	18	0.0	10.4	**89.6**
	NCD	CO^Maj b^ ≥ 3 + IR^Maj b^ > 1.5 only^f^	54	0.0	6.7	**93.3**
		CO^Maj b^ ≥ 3	42	0.0	3.1	**96.9**
		IR^Maj b^ > 1.5 only	12	0.0	4.7	**95.3**

Proportions in bold represent agreement between the predicted classification and the true classification

*RCD* Reference Chemicals Databases, *NCD* European New Chemicals Database, *CO* corneal opacity, *CR* conjunctiva redness, *CC* conjunctiva chemosis, *IR* iritis, *NA* not applicable

^a^None of the chemicals in this group was classified based on the driver 1 ≤ IR^Maj^ ≤ 1.5 only

^b^second (*n* = 3 or 4) or third (*n* = 5 or 6) highest mean score

^c^The largest of the second (*n* = 3 or 4) or third (*n* = 5 or 6) highest mean CR and CC scores

^d^Observed any time during the observation period (before day 21)

^e^Sample size is too small to perform a separate resampling analysis for this driver

^f^Individual animal data only accessible for the mean scores of days 1–3. Information on persistence of effects is only available in the form of comments, often not specifying in which tissues and in how many animals the persistence of effects was observed. Consequently, it was not possible to estimate the resampling probabilities for the chemicals that were classified based only on CO = 4 (observed any time during the observation period, before day 21) or persistence of a tissue effect. The resampling probabilities reported for the classification drivers CO^Maj^ ≥ 3 and IR^Maj^ > 1.5 should therefore not be considered as representative of the overall resampling probabilities for chemicals classified as Cat 1 in the NCD

## Discussion

For more than two decades, scientists have been trying to replace the regulatory Draize eye test by in vitro methods, but so far only partial replacement has been achieved. In order to better understand the reasons for this apparent lack of success, historical in vivo rabbit eye irritation data from three Reference Chemicals Databases (RCD), namely ECETOC, ZEBET and LNS, and from the European New Chemicals Database (NCD) were evaluated.

The analyses presented in this paper show that about 83 % of the chemicals that were registered in the NCD with valid data for the purpose of these analyses were not classified (No Cat). Furthermore, 10 % were classified as UN GHS/EU CLP Cat 2 (reversible effects on the eye/irritant to eyes) and 7 % as UN GHS/EU CLP Cat 1 (irreversible effects on the eye/serious eye damage). This prevalence is expected to be closer to the reality than what is observed in the RCD, which contains a higher number of classified chemicals, since the NCD contains all chemicals registered by multiple industry sectors since 1981, while the RCD contains a limited number of chemicals that were put together in databases mainly to support validation studies.

Differences in the endpoints driving classification were also observed between the RCD and NCD. For example, 65 % of the Cat 1 chemicals in the NCD were classified based on persistence of effects only, whereas this was 35 % for the RCD. Importantly, chemicals that were classified Cat 1 based only on persistence of effects in both the RCD and NCD have individual animal mean CO scores that are mostly below 3 (in 74/76 rabbits) and mean IR scores that are mostly below 1.5 (in 72/76 rabbits), i.e. below the CO and IR Cat 1 classification thresholds (Fig. 1). In fact, in the NCD, more than 25 % of the animals from this group of Cat 1 chemicals actually have mean CO scores below 1, while most of them in both the NCD and RCD have mean IR scores below 1, i.e. the CO and IR No Cat score ranges. This demonstrates that low tissue scores in the beginning of a study (days 1–3) are not predictive of the absence of persistent effects. It could be speculated that these chemicals induce eye irritation by different modes of action than those that induce immediate severe effects (e.g. through delayed effects or persistence of low-level effects). For this reason and because persistence of effects seems to play such an important role in the classification of a chemical as Cat 1 by the in vivo method, it may be necessary to have in vitro test methods capable of directly detecting persistence of effects. Certainly, it is known that the under-predictions for Cat 1 chemicals obtained with organotypic methods like BCOP and ICE, which were developed to detect immediate severe effects, are more likely for chemicals classified in vivo based only on persistence of effects than for those classified based on severity (ICCVAM 2006a, b, 2007). Two other organotypic methods, the Ex-Vivo Eye Irritation Test (EVEIT) based on isolated rabbit corneas (Spöler et al. 2007; Frentz et al. 2008) and the Porcine Corneal Ocular Reversibility Assay (PorCORA) based on isolated porcine corneas (Piehl et al. 2010, 2011), have been proposed to directly address reversibility/persistence of effects, but neither has yet undergone a formal validation study. Both these methods are based on the direct monitoring of the healing process in exposed excised corneas kept in culture over several days. This type of methods may be needed for the correct identification of Cat 1 chemicals and may significantly contribute in a testing strategy to the full replacement of the in vivo Draize eye test method.

Of note, a substantial proportion of the Cat 1 chemicals is currently being classified based only on persistence of conjunctiva effects (6.5 % (4/62) in the RCD and 9.3 % (12/129) in the NCD). More importantly, the majority of these chemicals are classified as Cat 1 based only on a CR and/or CC score of 1 at day 21 (all 4 chemicals from the RCD and 8 of the 12 from the NCD). In fact, the severity of CO (grade 1, 2, 3, or 4) and IR (grade 1 or 2) at day 21 for Cat 1 chemicals showing persistence of effects in at least one animal is more or less equally distributed over the grades, whereas for CR and CC, lower grades were observed more frequently, with the majority being scores equal to 1 (Table 4). This suggests that conjunctiva effects behave differently than corneal or iris effects, in that conjunctiva effects are mostly reversible by nature independently of the chemical tested, while iritis and especially corneal opacity are not. What is more, CR and CC are the only in vivo endpoints that consistently show scores greater than 0 for No Cat chemicals (Figs. 1, 2). CR in particular actually shows mean scores equal to or greater than 1 in 25.6 % (155/606) and 23.6 % (1,088/4,611) of the animals that were tested with No Cat chemicals in the RCD and in the NCD, respectively. Some conjunctiva redness may even be present in non-treated animals. It is therefore highly questionable to treat CR and CC in a similar way as CO and IR when it comes to classify chemicals as Cat 1 based on persistence of effects at day 21. Based on the data presented here, we believe that chemicals should not be classified as Cat 1 based on CR and/or CC scores at day 21 greater than 0 but less than the Cat 2 classification threshold (i.e. 0 < CR/CC < 2), in the absence of any other Cat 1-triggering effects. Indeed, CR and/or CC scores of less than 2 at day 21 should be considered as fully reversed conjunctiva effects and, for animal welfare reasons, animal studies should be stopped when CO and IR have fully reversed to scores equal to 0, and CR and CC have reached scores of less than 2. This is already the case with the U.S. EPA classification system, which considers CR and CC scores of less than 2 as fully cleared (U.S. EPA 1998, 2011). Considerations should therefore be given to a revision of UN GHS and EU CLP in the same direction.

It is also currently accepted and expressed in multiple regulations (EC 2008; OECD 2012a; UN 2013) that extreme CO scores should be irreversible by nature. However, in several cases of animals scored with a CO = 4 any time during the observation period (in at least 9 % of the animals), these extreme opacities were able to reverse to 0 by day 21 or even earlier (e.g. in the RCD, 9 animals scored with a CO = 4 reversed to CO = 0 by days 21 (2 animals), 11, 10, 7 (4 animals), and even 3). Considering the subjective scoring of the effects observed in the in vivo Draize eye test, it is debatable if such chemicals should be classified Cat 1 in the absence of any other Cat 1-triggering effects, i.e. with CO^Maj^ < 3, with IR^Maj^ ≤ 1.5, and without persistence of effects at day 21. Again, according to the U.S. EPA classification system, chemicals showing CO = 4 that reverses to 0 by day 21 or earlier are not classified as U.S. EPA Cat I if CO and IR revert to 0 and CR and CC revert to less than 2 by day 21 in all tested animals (U.S. EPA 1998, 2011). In fact, under UN GHS and EU CLP, Cat 1 is also associated with “irreversible effects on the eye”, being defined as “the production of tissue damage in the eye,… which is not fully reversible within 21 days of application”, while Cat 2 is associated with “reversible effects on the eye”, being defined as “the production of changes in the eye …, which are fully reversible within 21 days of application”. So, according to these definitions, such chemicals should indeed be classified as Cat 2, but currently a CO = 4 is taking precedence over the fact that the observed injury might be reversible by day 21. It is our opinion that UN GHS and EU CLP should be revised to clarify this situation, and it is our recommendation that if a study was continued after a CO = 4 was noted and it was shown that the observed effect was fully reversible within 21 days, such CO = 4 should not trigger a Cat 1 classification. Moreover, seeing that CO = 4 could be a highly subjective scoring that is not always linked to persistence of corneal opacity at day 21, it would probably be more correct to only terminate studies where CO = 4 is observed before day 21, without investigating the reversibility of the effect and accepting a Cat 1 classification, if such effects are observed in the majority of the animals tested, i.e. in 2 out of 3, 3 out of 4, 3 out of 5, or 4 out of 6 animals. This approach seems reasonable seeing that it would be identical to what is already used for the other severity based criteria CO^Maj^ and IR^Maj^.

In our analyses, iritis was found to rarely drive the classification of chemicals in vivo (both Cat 1 and Cat 2) on its own (<4 % of the chemicals from both NCD and RCD) (Tables 3, 5). In fact, when the iris shows scores above the classification cut-offs, these are almost every time accompanied by corneal opacity scores also above the classification cut-offs. Specifically, 95.7 % (44/46) of the chemicals in the RCD and 89.2 % (83/93) of the chemicals in the NCD that have 1 ≤ IR^Maj^ ≤ 1.5 (Cat 2 range) also have CO^Maj^ ≥ 1, and 88.9 % (8/9) of the chemicals in the RCD and 55.6 % (5/9) of the chemicals in the NCD that have IR^Maj^ > 1.5 (Cat 1 range) also have CO^Maj^ ≥ 3 and/or CO = 4. As such, effects on the iris are of lesser importance for classification of chemicals under the UN GHS/EU CLP classification systems. Therefore, it seems logical to deprioritise the development of new in vitro methods to predict iritis as well as the use of this endpoint as reference in the development of in vitro testing strategies.

Our analyses also show that the two most important endpoints that drive Cat 2 classification in both the RCD and NCD are conjunctiva redness and corneal opacity. These two endpoints together account for the classification of more than 95 % of the Cat 2 chemicals in both the RCD and NCD, which again demonstrates the lack of weight of iritis, but also of conjunctiva chemosis, in the identification of Cat 2 chemicals with the current in vivo Draize eye test method. Altogether, 80.9 % of the Cat 2 chemicals in the RCD and 74.7 % of those in the NCD are classified based on CR^Maj^ ≥ 2 (versus 51.1 % (RCD), and 24.7 % (NCD) based on CC^Maj^ ≥ 2), while 74.5 % of the Cat 2 chemicals in the RCD and 54.1 % of those in the NCD are classified based on CO^Maj^ ≥ 1. Interestingly, conjunctiva redness appears to be twice as important as corneal opacity for the classification of Cat 2 chemicals on their own since 23.4 % and 41.2 % of the Cat 2 chemicals in the RCD and NCD, respectively, are classified based on CR^Maj^ ≥ 2 without enough corneal opacity to generate a classification, while only 10.6 % and 20.1 % of the Cat 2 chemicals in the RCD and NCD, respectively, are classified based on CO^Maj^ ≥ 1 without enough conjunctiva redness to generate a classification. This indicates that there will also be a need for in vitro methods that can identify conjunctiva effects, and especially conjunctiva redness, to fully replace the in vivo Draize eye test if the animal data continue to be interpreted as currently expressed in UN GHS and EU CLP and the classifications obtained thereafter continue being used as reference in the evaluation/validation of alternative methods. The Hen’s Egg Test–Chorioallantoic Membrane (HET-CAM) (Luepke 1985; de Silva et al. 1992; Gilleron et al. 1996; Spielmann et al. 1996) and similar methods like the Chorioallantoic Membrane Vascular Assay (CAMVA) (Bagley et al. 1994) have been proposed to provide information on conjunctiva effects in vivo due to the similarity of the CAM to the conjunctiva. Both HET-CAM and CAMVA underwent multiple international validation studies (Bagley et al. 1992b, 1999b; Spielmann et al. 1993, 1996, 1997; Brantom et al. 1997; Ohno et al. 1999; Hagino et al. 1999). More recently, HET-CAM was also formally validated by the Interagency Coordinating Committee on the Validation of Alternative Methods (ICCVAM) for the UN GHS classification system, but it was not considered useful at that time to be used for regulatory purposes for the evaluation of the serious eye damage/eye irritation potential of chemicals, due to the lack of sufficient data for Cat 2 chemicals (ICCVAM 2010). Considering the present findings, it might be useful to reconsider the usefulness of HET-CAM/CAMVA for inclusion in an in vitro eye irritation testing strategy and evaluate if the additional data generated since 2009 and/or any modifications to its protocol and/or prediction model can be useful for that purpose. It is worth mentioning that ICCVAM recommended in particular that additional data be collected on eye irritants (Cat 2) to more adequately characterise the usefulness of the HET-CAM. Test methods using reconstructed human tissues (RhT) modelling the corneal epithelium, like the EpiOcular™ Eye Irritation Test (EIT) (Kaluzhny et al. 2011; Pfannenbecker et al. 2013) or the SkinEthic™ Human Corneal Epithelium (HCE) test (Van Goethem et al. 2006; Cotovio et al. 2007, 2010; Alépée et al. 2013), may also be relevant for assessing conjunctiva epithelial responses using cytotoxicity as an endpoint (OECD 2010). An EURL ECVAM/Cosmetics Europe prospective validation study on EpiOcular™ EIT and SkinEthic™ HCE to evaluate their usefulness and limitations for discriminating non-classified materials versus eye irritants/chemicals inducing serious eye damage (Cat 2/Cat 1) is currently ongoing (Freeman et al. 2010). Considering the small prevalence of eye irritants and chemicals inducing serious eye damage (Table 1), these methods could be very important for the identification of chemicals not requiring classification in a non-animal testing strategy.

The variability of the responses observed between rabbits from historical data normally used as reference in validation studies is also recognised as a challenging factor for the success of such studies (Scott et al. 2010) and may ultimately hinder the acceptance of in vitro methods. Statistical resampling of available historical in vivo Draize eye test data was therefore performed to further evaluate the test method’s within-test variability. Our analyses show that about 12 % of the NCD chemicals classified as Cat 2 by the in vivo Draize eye test method and at least 11 % of those classified as Cat 1 could in fact be equally identified as No Cat and as Cat 2, respectively, simply due to the inherent within-test variability of this reference method. Indeed, the resampling analyses show that the within-test variability of the Draize eye test is quite high for the Cat 2 subgroup that was classified based on CR^Maj^/CC^Maj^ ≥ 2 (without CO^Maj^ ≥ 1), with under-classification rates as No Cat of 6 % (RCD) and 10 % (NCD), and for the Cat 1 subgroups that were classified based on CO = 4 only or persistence of effects only, with under-classification rates as Cat 2 of about 13 % in the RCD. The under-prediction rate for the Cat 1 subgroup that was classified based on persistence of effects only increased to 22 % when only liquids were considered. These three classification drivers largely contribute to the observed overall under-prediction probability of Cat 2 chemicals as No Cat of 4 % (RCD) and 12 % (NCD) and to an overall under-prediction probability of Cat 1 chemicals as Cat 2 of at least 11 %. A similar analysis performed on data of 723 rabbits tested in 181 studies found under-classification probabilities ranging from 4.30 to 13.24 %, with the estimated under-classification rate also being higher for chemicals classified based on persistence only (Haseman et al. 2005). The under-prediction probabilities for the chemicals classified as Cat 2 based on 1 ≤ CO^Maj^ < 3 and for the chemicals classified as Cat 1 based on CO^Maj^ ≥ 3 were generally smaller than those obtained for the other classification drivers, ranging from 1 to 6 %, depending on the database, the classification driver and the physical state of the chemical. This indicates that corneal opacity is the most consistent tissue effect observed in the Draize eye test and probably also the easiest one (least subjective) to score. Not only is corneal opacity the endpoint showing lower variability, it is also one of those bearing most weight in the classification of chemicals in vivo. It is therefore not surprising that most of the in vitro methods that have been developed to date address this specific endpoint, which seems an appropriate strategy to follow. Finally, as opposed to classified chemicals, the within-test variability for No Cat chemicals was very low; indeed, the resampling probability to consistently identify these chemicals as No Cat is more than 99 % in both RCD and NCD. The No Cat chemicals rarely induce corneal opacity or iritis, and often also no conjunctiva chemosis. The only effect that was observed somewhat more frequently for these chemicals in the RCD and NCD was some conjunctiva redness, as already mentioned above, which somehow disputes the systematic use of this endpoint to classify chemicals as Cat 2 using a cut-off score of 2.

It is important to note that the current analyses only took into account the within-test variability of the Draize eye test method. Weil and Scala (1971) and Cormier et al. (1996) studied the variability of responses of nine substances tested in up to 24 laboratories to address both the within- and the between-laboratory variability of the test method. The authors showed that the Draize eye test can produce quite variable results among laboratories as well as within certain laboratories. Certain materials were rated as the most irritant tested by some laboratories and, contrariwise, as the least irritant by others. The authors suggested that the primary reason for the observed extreme variation between laboratories is in the reading of reactions. Unconscious bias or definite tendencies to over- or under-read reactions or misinterpret the meaning of descriptive terms may have accounted for that. In addition, variation in interpretation and performance of the procedures was also reported as a component for the observed between-laboratory variability (Weil and Scala 1971). Marzulli and Ruggles (1973) also studied the between-laboratory variability of seven materials tested in ten laboratories and confirmed the findings from Weil and Scala. Although the authors suggest that laboratories were able in most cases to distinguish chemicals inducing serious eye damage/eye irritants from non-irritants, statistically significant differences were found between collaborators with regard to the tissue readings. Thus, if the within- and between-laboratory variability of the Draize eye test were to be added to the within-test variability reported here, it is expected that the overall variability of the in vivo method would even increase. For all the reasons above, the in vivo under-prediction rates reported in this study should be taken into account when evaluating alternative methods/strategies to replace the animal test.

Lovell (1996) performed a principal component analysis on Draize eye tissue scores obtained 24, 48, and 72 h after instillation of a test substance into the rabbit eye for fifty-five chemicals from the ECETOC database and found that the first component, which was highly correlated with the maximum individual weighted scores (used to calculate the modified maximum average score, MMAS), could already explain 76 % of the variability. He concluded that there was only limited evidence for differential responses of different tissues. This was not confirmed in our study, the main reason being that Lovell focused only on the in vivo scores obtained in the first 3 days of the study, while we considered the full in vivo data necessary to classify according to UN GHS/EU CLP decision criteria. Weighed Draize scores, like (M)MAS, are irrelevant for regulatory purposes as they omit important information like the persistence/reversibility of effects. Indeed, chemicals with intermediate (M)MAS can either correspond to a Cat 1 or a Cat 2 classification, while chemicals with high (M)MAS are not necessarily classified as Cat 1. Our analyses indicate that under current UN GHS/EU CLP classification rules, the (M)MAS should not be used as benchmark for the evaluation of in vitro test data. Instead, the focus should be on the classification drivers described in this paper.

## Conclusions

In vitro methods partially replacing the in vivo Draize eye test for the classification of chemicals inducing serious eye damage according to UN GHS (Cat 1) are accepted by the OECD since 2009 (BCOP TG 437, ICE TG 438) (OECD 2013a, b) and 2012 (FL TG 460) (OECD 2012b). In addition, the BCOP and ICE test methods are also accepted since 2013 to identify chemicals that do not require classification under UN GHS (and consequently also under EU CLP) (No Cat) (OECD 2013a, b). Finally, the CM (Hartung et al. 2010) and the STE (Takahashi et al. 2008, 2009; Sakaguchi et al. 2011) have been endorsed as scientifically valid for their limited applicability domains (ESAC 2009; ICCVAM 2013), and are currently in the process of regulatory adoption by the OECD for the identification of chemicals inducing serious eye damage (Cat 1) as well as chemicals not requiring a classification for serious eye damage/eye irritation (No Cat). Further development and/or evaluation of alternative methods are, however, still required to fill the gaps identified in this paper, i.e. persistence/reversibility of effects and identification of Cat 2. Considering the importance of conjunctiva effects for the classification of Cat 2 chemicals (43 % in the NCD and 26 % in the RCD) and of persistence of corneal effects for the classification of Cat 1 chemicals (53 % in the NCD and 26 % in the RCD), focus and efforts may still have to be given to further development and evaluation/validation of alternative methods/strategies capable of correctly identifying these effects. Several other test methods, e.g. the EpiOcular™ EIT (Kaluzhny et al. 2011; Pfannenbecker et al. 2013), the SkinEthic™ HCE (Van Goethem et al. 2006; Cotovio et al. 2007, 2010; Alépée et al. 2013), the Ocular Irritection^®^ assay, the PorCORA (Piehl et al. 2010, 2011) or the EVEIT (Spöler et al. 2007; Frentz et al. 2008), are currently undergoing validation or are in an advanced status of development/optimisation and should soon be accepted for use in a regulatory environment. With all these methods becoming available, it should now be possible to explore the development of highly predictive testing strategies capable of fully replacing the animal test. For example, considering currently available data, it is likely that test methods such as BCOP, ICE, HET-CAM, STE, and RhT-based assays like EpiOcular™ EIT or SkinEthic™ HCE, although not directly addressing conjunctiva effects (apart maybe from HET-CAM), may be able to correctly predict the irritation potential of chemicals classified in vivo based only on conjunctiva effects, when used to discriminate non-classified (No Cat) from all classified (Cat 2 + Cat 1) chemicals. Priority should therefore be given in the near future to the development, optimisation and/or validation of in vitro test methods capable of discriminating reversible from irreversible effects.

In order to develop adequate, stand-alone strategies for the assessment of serious eye damage/eye irritation with alternative methods, it is important to understand which endpoints drive UN GHS/EU CLP classification in the in vivo reference method. The endpoint that drives classification is also an important factor that needs to be considered when selecting representative candidate chemicals for the development, validation, and regulatory acceptance of in vitro alternatives. It is important not only to select reference chemicals covering the essential drivers of irritation as described in this paper, i.e. severe corneal lesions, persistence of corneal effects in the absence of severe lesions, and conjunctiva effects, but also only those having high-quality in vivo data, resulting in less questionable and more reproducible classifications (De Wever et al. 2012; Barroso et al. 2013). At the same time, the UN GHS and EU CLP decision criteria to classify chemicals based on Draize eye test data should be critically reviewed. The resampling analyses presented here show that the Draize eye test is prone to high misclassification errors. Importantly, these misclassification errors are unidirectional towards lower classifications, which demonstrates that the way the Draize eye test data are interpreted is very conservative and may over-predict the true irritation potential of chemicals; indeed, comparative studies between rabbit and man have shown that the Draize eye test is likely to overestimate the effects in man (Roggeband et al. 2000). The following decision criteria, in particular, should therefore be reconsidered: (1) the biological relevance of a threshold of 2 for classifying chemicals as Cat 2 based on CR should be reassessed considering that a significant proportion of No Cat chemicals show mean CR scores equal to or greater than 1 and that some CR may even be present in non-treated animals; (2) CR and/or CC scores of less than 2 at day 21 should be considered as fully reversed conjunctiva effects and should therefore not drive a Cat 1 classification in the absence of any other Cat 1 triggering effects; (3) CO = 4 that fully reverse within 21 days should not trigger a Cat 1 classification in the absence of any other Cat 1 triggering effects; and (4) studies where CO = 4 is observed should only be terminated before day 21, without investigating the reversibility of the effect and accepting a Cat 1 classification, if such effects are observed in the majority of the animals tested, i.e. in 2 out of 3, 3 out of 4, 3 out of 5 or 4 out of 6 animals.

In order to be successful in the task of replacing animal testing, it is of utmost importance to recognise that animal reference methods are not perfect and to investigate and consider their limitations in the assessment of in vitro alternatives. Thus, the uncertainties associated with these animal tests and their benchmark data should be taken into account when evaluating/validating alternative methods and/or strategies developed to replace them. In this paper, we show that due to animal-to-animal within-test variability, there is an overall probability of at least 11 % that chemicals classified as Cat 1 by the in vivo Draize eye test under UN GHS/EU CLP could be equally identified as Cat 2. We also show that due to this inherent within-test variability, about 12 % of the Cat 2 chemicals could be equally identified as No Cat by the in vivo Draize eye test. Importantly, these probabilities may certainly increase if the within- and between-laboratory variabilities are also considered (Weil and Scala 1971; Marzulli and Ruggles 1973; Cormier et al. 1996). The in vivo misclassification estimates presented here should therefore be acknowledged in the development of alternative test methods and testing strategies for serious eye damage/eye irritation and should also be considered when defining acceptance levels of false negatives and false positives during their evaluation/validation and, most importantly, during their regulatory acceptance.

## List of abbreviations

BCOP: Bovine Corneal Opacity and Permeability
CAMVA: Chorioallantoic Membrane Vascular Assay
Cat 1: UN GHS/EU CLP classification for chemicals causing irreversible effects on the eye/serious damage to the eye
Cat 2: UN GHS/EU CLP classification for chemicals causing reversible effects on the eye/eye irritation
CC: Conjunctiva chemosis
CC^Maj^: Corresponds to the second highest (for studies with 3 or 4 animals) or the third highest (for studies with 5 or 6 animals) of the mean CC scores (of gradings at 24, 48, and 72 h)
CO: Corneal opacity
CO^Maj^: Corresponds to the second highest (for studies with 3 or 4 animals) or the third highest (for studies with 5 or 6 animals) of the mean CO scores (of gradings at 24, 48, and 72 h)
CR: Conjunctiva redness
CR^Maj^: Corresponds to the second highest (for studies with 3 or 4 animals) or the third highest (for studies with 5 or 6 animals) of the mean CR scores (of gradings at 24, 48, and 72 h)
Ex-ECB: Ex-European Chemicals Bureau
ECETOC: Eye Irritation Reference Chemicals Data Bank
EIT: Eye Irritation Test
EU CLP: European Union Regulation on Classification, Labelling and Packaging of chemicals implementing UN GHS in the EU
EVEIT: Ex-Vivo Eye Irritation Test
FL: Fluorescein Leakage
HCE: Human Corneal Epithelium
HET-CAM: Hen’s Egg Test–Chorioallantoic Membrane
ICCVAM: Interagency Coordinating Committee on the Validation of Alternative Methods
ICE: Isolated Chicken Eye
IR: Iritis
IR^Maj^: Corresponds to the second highest (for studies with 3 or 4 animals) or the third highest (for studies with 5 or 6 animals) of the mean IR scores (of gradings at 24, 48, and 72 h)
LNS: Laboratoire National de la Santé
NCD: European New Chemicals Database
No Cat: Chemicals not classified for serious eye damage/eye irritation under UN GHS/EU CLP
OECD: Organisation for Economic Co-operation and Development
PorCORA: Porcine Corneal Ocular Reversibility Assay
RCD: Reference Chemicals Databases
RhT: Reconstructed human Tissue
UN GHS: United Nations Globally Harmonized System of Classification and Labelling of Chemicals
